# Organ-Specific Clinicopathological Features That Are Associated With Post-Relapse Survival of Metastatic Breast Cancer in Japanese Women: A Multicenter Cohort Study

**DOI:** 10.14740/wjon2662

**Published:** 2025-12-17

**Authors:** Yoichi Koyama, Yoshiya Horimoto, Akimitsu Yamada, Kazutaka Narui, Shinya Yamamoto, Hiroshi Kaise, Kimito Yamada, Takashi Ishikawa

**Affiliations:** a Department of Breast Surgical Oncology, Tokyo Medical University Hospital, Tokyo, Japan; b Department of Breast Oncology, Juntendo University Faculty of Medicine, Tokyo, Japan; c Department of Gastroenterological Surgery, Yokohama City University Graduate School of Medicine, Kanagawa, Japan; d Department of Breast and Thyroid Surgery, Yokohama City University Medical Centre, Kanagawa, Japan; e Department of Breast Surgery, Yokohama Rosai Hospital, Kanagawa, Japan; f Department of Breast Surgical Oncology, Tokyo Medical University Hachioji Medical Centre, Tokyo, Japan

**Keywords:** Breast neoplasms, Neoplasm metastasis, Molecular typing, Prognosis, Brain neoplasms, Retrospective studies

## Abstract

**Background:**

Metastatic breast cancer (MBC) presents heterogeneous clinical behavior depending on the metastatic site and molecular subtype. However, only a few studies have directly compared prognostic outcomes by the organ of initial distant recurrence. This multi-institutional retrospective study aimed to clarify the clinical and biological characteristics of MBC based on cases with single-organ metastasis at the time of initial MBC diagnosis, using real-world data from Japanese patients.

**Methods:**

We retrospectively analyzed 309 Japanese women treated for early-stage breast cancer at six institutions between 2007 and 2021, who developed distant recurrence confined to a single organ (brain, lungs, liver, or bones) as the first site of metastasis. Patients with multi-organ metastases at initial recurrence were excluded.

**Results:**

The median distant metastasis-free survival and post-relapse survival (PRS) were 29.4 and 39.7 months, respectively. PRS was longest in patients with bone recurrence, followed by those with lung and liver recurrence, and shortest in those with brain recurrence (P < 0.001). Bone and lung metastases were more frequently associated with hormone receptor-positive tumors, whereas liver and brain metastases were enriched for human epidermal growth factor receptor 2 (HER2)-negative and triple-negative tumors. The multivariable Cox model identified older age, triple-negative subtype, and symptomatic recurrence as independent poor prognostic factors, while liver and brain metastases were also independently associated with shorter PRS. Site-specific analyses revealed that triple-negative subtype and symptomatic presentation were consistent markers of poor prognosis across most metastatic sites.

**Conclusions:**

Based on a single-organ metastasis cohort, this study identified distinctive clinicopathological features and survival outcomes according to the site of initial distant recurrence. Brain metastases were associated with the poorest outcomes, highlighting the need for improved risk stratification and treatment strategies.

## Introduction

Breast cancer is the most common cancer among women worldwide, and while its mortality has significantly decreased in high-income countries such as the United States, it has continued to increase in Japan [1]. While many patients have favorable outcomes, with 5-year relative survival rates exceeding 90% in Japanese patients [2], the development of recurrence or distant metastasis leads to a marked decrease in survival, with the 5-year survival rate for those with distant metastases dropping to around 40% [2]. Therefore, improving the prognosis of metastatic breast cancer (MBC) remains an important clinical challenge.

Among patients who develop MBC following curative treatment of primary breast cancer, common sites of metastasis include the bones, liver, lungs, and brain [3], and metastases in these organs are known to impact the behavior and prognosis of MBC. For instance, patients with bone metastasis, unaccompanied by visceral involvement, reportedly have a median overall survival of about 50 months, indicating a relatively favorable prognoses based on a French multicenter cohort study [4]. Conversely, prognoses become extremely poor once brain metastasis occurs, with median survival typically ranging from 12 to 18 months in cohorts from the United States [5]. These observations suggest that the site of distant metastasis plays a crucial role in determining clinical outcomes in MBC. However, most existing studies have analyzed cohorts that included a substantial proportion of patients with metastases to multiple organs at diagnosis. As a result, it remains difficult to isolate the prognostic implications of individual metastatic sites. A few population-based studies using the Surveillance, Epidemiology, and End Results (SEER) database in the United States have shown that patients with bone-only metastasis have the most favorable survival, while those with brain-only metastasis have the poorest outcomes [6-8]. Similarly, a nationwide Danish registry study demonstrated a similar trend [9]. Nonetheless, these studies were limited by a lack of detailed information on treatment and disease progression, and they primarily focused on Western populations. Given the possibility of racial differences in metastatic patterns and outcomes, further investigation targeting Asian cohorts, particularly Japanese patients, is warranted.

A focus on patients with single-organ metastasis at the time of initial recurrence holds several important advantages. It allows for the evaluation of the biological and prognostic characteristics specific to each metastatic site while minimizing the confounding effects of additional metastatic spread. This approach is also critical for elucidating the relationship between metastatic organ preference and tumor molecular subtypes. Furthermore, as the site of initial metastasis strongly influences therapeutic decisions, site-specific prognostic data can provide valuable support for individualized treatment planning. Such insights may also facilitate risk stratification based on organotropism and contribute to the broader understanding of metastatic mechanisms. Therefore, we aimed to use real-world data to elucidate the biological features of MBC in Japanese patients, with a particular focus on the prognostic relevance of single-organ metastasis at initial recurrence.

## Materials and Methods

### Patient cohorts

**Figure 1:**
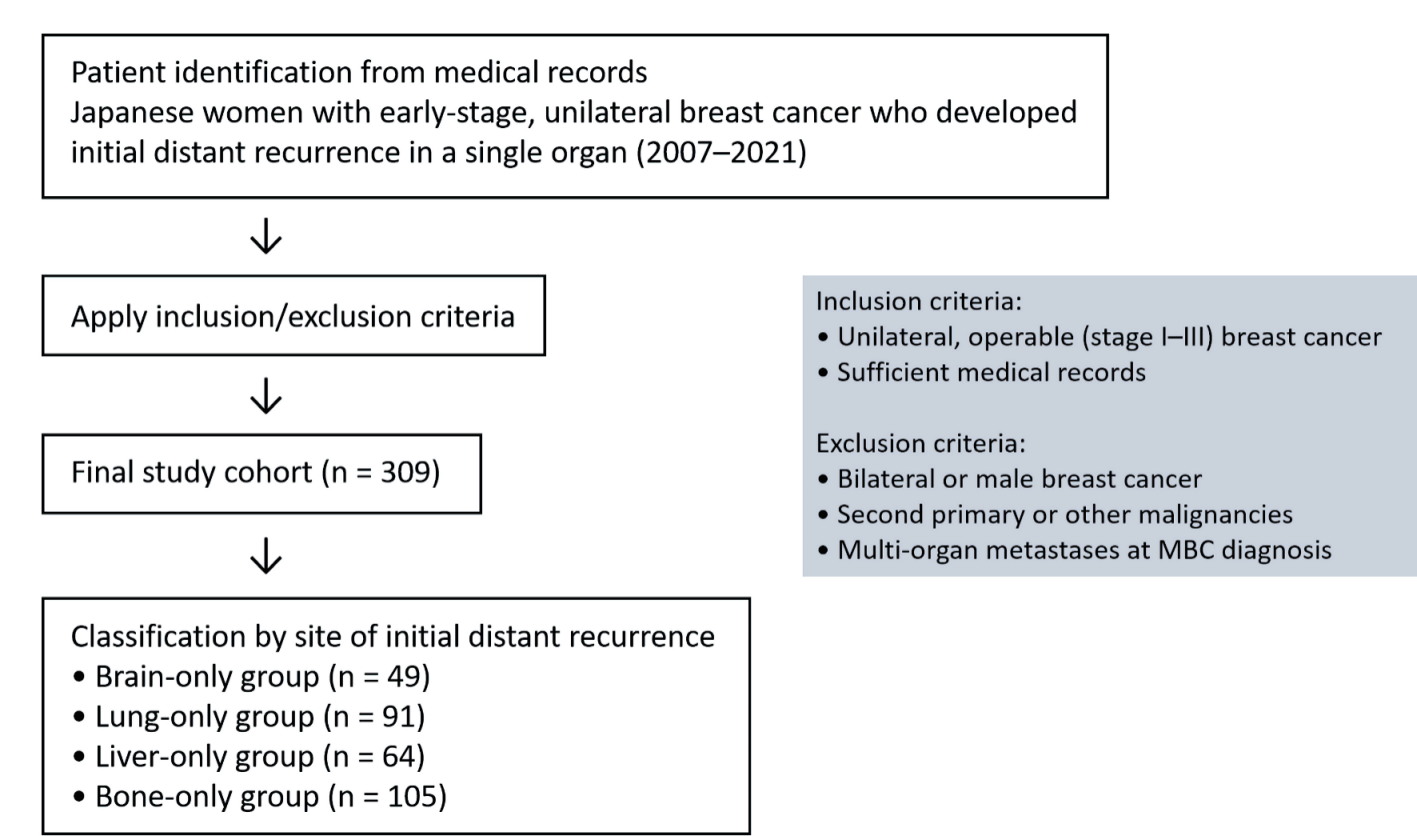
Flow diagram of study design. A flow diagram of the study design, including inclusion and exclusion criteria, is shown. MBC: metastatic breast cancer.

This was a multi-institutional, retrospective cohort study involving Japanese female patients with primary early-stage breast cancer treated at six institutions between January 2007 and December 2021. The participating institutions were Tokyo Medical University Hospital, Tokyo Medical University Hachioji Medical Center, Yokohama City University Graduate School of Medicine, Yokohama City University Medical Center, Yokohama Rosai Hospital and Juntendo University Hospital. The study design is summarized in Figure 1. Clinical data, including patient background and tumor characteristics, were retrospectively collected from medical records. For this analysis, we focused on patients who developed distant recurrence limited to a single organ - either the brain, lungs, liver, or bones - as the initial metastatic site. The following patients were excluded from this analysis: patients with disseminated carcinomatosis of the bone marrow at the time of initial bone metastasis; non-brain central nervous system involvement (such as spinal cord, pituitary gland, paranasal sinus, or leptomeningeal carcinomatosis); or pleural involvement without parenchymal lung metastasis. Patients with liver-only metastasis presenting with visceral crisis, defined according to the Advanced Breast Cancer (ABC) guidelines as rapidly increasing bilirubin levels, were also excluded [10]. After applying these criteria, a total of 309 patients were included and classified into four groups according to the site of initial distant recurrence: brain (n = 49), lungs (n = 91), liver (n = 64), and bones (n = 105). Treatment strategies before and after recurrence varied across participating institutions.

Post-recurrence survival (PRS) was defined as the time from the diagnosis of MBC to death from any cause or the last follow-up. The primary objective of this study was to compare PRS according to the site of initial distant recurrence. Additionally, exploratory analyses were performed to identify clinicopathological factors associated with prognosis in each metastatic site.

This study was approved by the Ethics Committee of Tokyo Medical University (approval number: T2022-0044) and the Institutional Review Board of each participating institution. Owing to the retrospective nature of the study, the requirement for informed consent was waived. Patients could review the research plan on the hospitals’ websites and were given the option to opt out of the study at any time. This study was conducted in compliance with the ethical standards of the responsible institution on human subjects as well as with the Helsinki Declaration.

### Pathological assessment

All pathological reports were retrospectively reviewed. Pathological assessments across institutions were performed uniformly based on the following criteria. Histopathological diagnosis followed the World Health Organization (WHO) classification, and tumor staging was based on the Union for International Cancer Control (UICC) TNM classification system. Nuclear grade was evaluated using the modified Bloom-Richardson system [11]. The Ki67 labeling index was assessed in a hotspot and categorized as low (≤ 20%) and high (> 20%). Estrogen receptor (ER) and progesterone receptor (PR) were considered positive when > 1% of tumor cell nuclei were stained. Human epidermal growth factor receptor 2 (HER2) was judged as positive if > 10% of the tumor cells exhibited strong staining of the entire cell membrane or if *HER2/neu* gene amplification was confirmed using fluorescence *in situ* hybridization. Molecular subtypes were classified as luminal (ER and/or PR positive, HER2 negative), luminal-HER2 (ER and/or PR positive, HER2 positive), HER2-enriched (ER and PR negative, HER2 positive), and triple-negative (ER, PR, and HER2 negative; triple-negative breast cancer (TNBC)).

### Statistical analyses

All statistical analyses were conducted using the IBM SPSS Statistics, Version 29 software package (IBM Corp., Armonk, NY, USA). Continuous and categorical variables were compared between cohort groups by using Kruskal-Wallis and Pearson’s Chi-square tests, respectively. Patient follow-up was monitored until October 2023, and prognostic analysis was conducted in November 2023. Kaplan-Meier survival curves were used to estimate the median PRS (95% confidence interval (CI)), which were compared using the log-rank test. Hazard ratios (HRs) and 95% CIs for factors potentially influencing PRS were estimated using univariate and multivariable Cox proportional hazards models. For the multivariable Cox regression analysis, variables were selected based on their clinical relevance, including age, Ki67 labeling index, molecular subtype, organ of initial distant recurrence, and primary trigger for MBC diagnosis. All tests were two-tailed, and P values < 0.050 were considered indicative of statistical significance.

## Results

### Clinical and pathological features according to organ of initial distant recurrence organ

The clinicopathological features of the 309 patients included in this study are compared according to the site of initial distant metastasis in Table 1. The median ages at primary breast cancer and MBC diagnoses were 58 (range: 21 - 89) and 61 (range: 29 - 90) years, respectively, and there were no differences among metastatic sites. Over 80% of patients had clinical stage II or higher diseases, and nearly 70% received perioperative chemotherapy. Notably, the brain-only group had a more advanced clinical stage, whereas a significantly higher proportion of patients in this group had an earlier pathological stage compared to the other groups (P = 0.004). Among the four metastatic sites, high nuclear grade tumors were significantly less frequent in the bone-only group (P < 0.001), while the lung-only group showed a tendency toward higher Ki67 labelling index (P = 0.002). The distribution of molecular subtypes also showed a significant difference by metastatic site. The bone-only group was predominantly composed of luminal-type tumors (77%), whereas the brain-only group had a lower proportion of luminal type and a higher proportion of HER2-enriched and TNBC (P < 0.001). Regarding the timing of recurrence, the bone-only group had the longest distant metastasis-free survival (DMFS) (37 months), while the brain-only group had the shortest (14 months). Kaplan-Meier curves for DMFS according to the site of metastasis are shown here (Supplementary Material 1, wjon.elmerpub.com). As for the diagnostic triggers, the brain-only group was overwhelmingly diagnosed due to subjective symptoms such as dizziness, accounting for 96% of cases (P < 0.001). In contrast, lung and liver recurrences were most frequently detected through routine postoperative surveillance, such as imaging or tumor marker monitoring (mainly carcinoembryonic antigen (CEA) and cancer antigen 15-3 (CA15-3)). The bone-only group showed an intermediate pattern between these two. Considering the marked difference in diagnostic triggers among metastatic sites, we further calculated DMFS focusing on only patients who had subjective symptoms (n = 123; 42% of all patients) (not shown). For those patients, median DMFS were as follows: brain-only, 12.8 months (range: 1 - 154); lung-only, 14.8 (range: 6 - 61); liver-only, 21.6 (range: 6 - 134); and bone-only, 31.6 (range: 2 - 167). Although the order of median DMFS values changed slightly, the overall pattern across metastatic sites remained similar.

**Table 1:** Patients’ Clinical and Pathological Background at Baseline

Baseline factors	All (n = 309)	Brain-only (n = 49)	Lung-only (n = 91)	Liver-only (n = 64)	Bone-only (n = 105)	P value
Age at PBC diagnosis (years), median (range)	58 (21 - 89)	58 (27 - 84)	59 (21 - 89)	56 (30 - 86)	58 (31 - 77)	0.575
Age at MBC diagnosis (years), median (range)	61 (29 - 90)	59 (29 - 84)	62 (29 - 90)	59 (34 - 87)	61 (35 - 81)	0.728
Histological type						
Invasive ductal carcinoma	266 (86%)	45 (92%)	76 (84%)	58 (91%)	87 (83%)	0.276
Others	43 (14%)	4 (8%)	15 (16%)	6 (9%)	18 (17%)	
Clinical disease stage of PBC						
Stage I	54 (17%)	4 (8%)	16 (18%)	13 (20%)	21 (20%)	0.288
Stage II/III	255 (83%)	45 (92%)	75 (82%)	51 (80%)	84 (80%)	
Pathological disease stage of PBC						
Stage 0/I	67 (21%)	20 (40%)	19 (21%)	11 (17%)	17 (16%)	0.004
Stage II/III	242 (78%)	29 (60%)	72 (79%)	53 (83%)	88 (84%)	
Nuclear grade						
Grade 1/2	141 (65%)	11 (48%)	33 (53%)	30 (59%)	67 (83%)	< 0.001
Grade 3	76 (35%)	12 (52%)	29 (47%)	21 (41%)	14 (17%)	
Unknown	92	26	29	13	24	
Ki67 labeling index						
Low (≤ 20%)	62 (27%)	8 (24%)	9 (12%)	20 (41%)	25 (35%)	0.002
High (> 20%)	165 (73%)	26 (76%)	64 (88%)	29 (59%)	46 (65%)	
Unknown	82	15	18	15	34	
Molecular subtypes						
Luminal	181 (59%)	14 (29%)	47 (52%)	39 (61%)	81 (77%)	< 0.001
Luminal-HER2	32 (10%)	7 (14%)	7 (8%)	11 (17%)	7 (7%)	
HER2-enriched	30 (10%)	13 (27%)	11 (12%)	3 (5%)	3 (3%)	
Triple-negative	66 (21%)	15 (31%)	26 (29%)	11 (17%)	14 (13%)	
Adjuvant chemotherapy for PBC						
Yes	219 (71%)	42 (86%)	55 (60%)	46 (72%)	76 (72%)	0.017
Neoadjuvant	143	32	37	34	40	
Adjuvant	76	10	18	12	36	
No	90 (29%)	7 (14%)	36 (40%)	18 (28%)	29 (28%)	
DMFS (months), median (range)	29.4 (1 - 198)	13.5 (1 - 198)	34.4 (1 - 172)	26.1 (2 - 134)	37.0 (2 - 167)	0.004
Primary trigger for MBC diagnosis						
Subjective symptoms	123 (42%)	44 (96%)	13 (15%)	14 (23%)	52 (51%)	< 0.001
Regular surveillance	172 (58%)	2 (4%)	75 (85%)	46 (77%)	49 (49%)	
Unknown	14	3	3	4	4	

PBC: primary breast cancer; MBC: metastatic breast cancer; HER2: human epidermal growth factor receptor 2; DMFS, distant metastasis free survival.

### Comparison of PRS by site of initial distant metastasis and identification of prognostic factors

**Figure 2:**
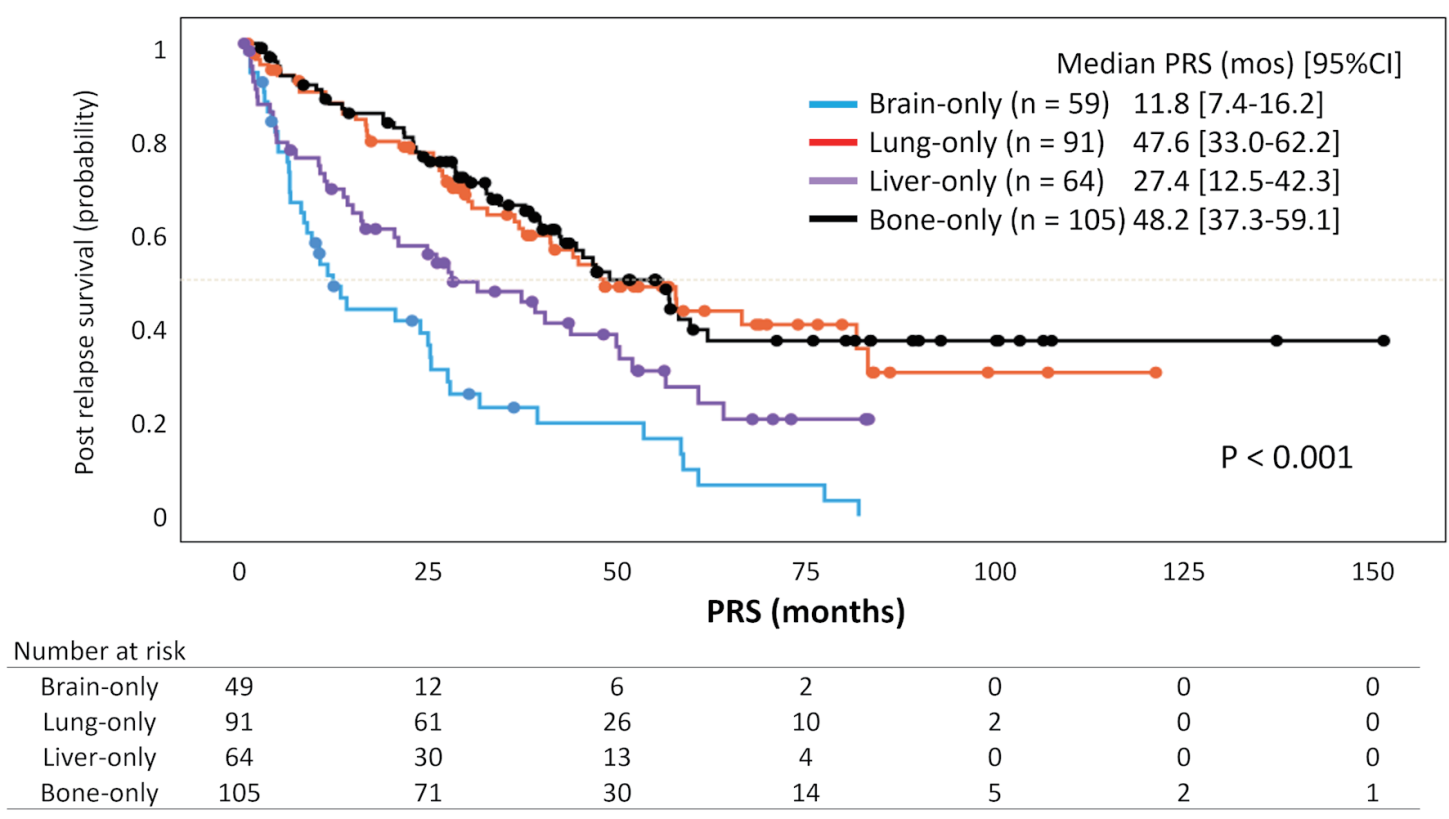
Kaplan-Meier curves for post-relapse survival according to organs of initial distant recurrence. The curves, stratified by the organs of initial distant recurrence, represent survival outcomes observed over a median follow-up period of 62.9 months (95% CI: 57.4 - 69.9). The blue, red, purple, and black curves correspond to brain-only, lung-only, liver-only, and bone-only groups, respectively. PRS: post-relapse survival; CI: confidence interval.

As of the time of this analysis, 177 patients had been confirmed to have died due to breast cancer. The median PRS for all patients was 39.7 months (95% CI: 32.3 - 47.1). First, Kaplan-Meier curves for PRS based on the organs of initial distant recurrence were drawn (Fig. 2). Patients with bone or lung metastases had the longest PRS, with median values of 48.2 months (95% CI: 37.3 - 59.1) and 47.6 months (95% CI: 33.0 - 62.2), respectively, followed by those with liver metastases at 27.4 months (95% CI: 12.5 - 42.3), whereas patients with brain metastases had the shortest PRS at 11.8 months (95% CI: 7.4 - 16.2) (P < 0.001).

Next, we investigated clinicopathological features relating to PRS. Multivariable Cox regression analysis revealed that older age at MBC diagnosis, TNBC and the presence of subjective symptoms as the primary trigger for MBC diagnosis were independent predictive factors for shorter PRS (P < 0.001, P < 0.001 and P = 0.003, respectively) (Table 2). Moreover, patients who developed brain or liver metastasis had significantly shorter PRS, compared to those with bones (median PRS: brain, 11.8 months; liver, 27.4 months; bone, 48.2 months; both P < 0.001).

**Table 2:** Factors Relating With Post-Relapse Survival (N = 309)

Clinicopathological factors	N	Univariate analysis			Multivariable analysis		
		HR	95% CI	P value	HR	95% CI	P value
Age at MBC diagnosis	309	1.01	1.00 - 1.03	0.020	1.03	1.01 - 1.04	< 0.001
Menopausal status at MBC diagnosis							
Premenopausal	78	Reference					
Postmenopausal	231	1.07	0.75 - 1.52	0.719			
Histological type							
Invasive ductal carcinoma	266	Reference					
Others	43	1.29	0.86 - 1.94	0.213			
Nuclear grade							
Grade 1/2	141	Reference					
Grade 3	76	2.27	1.56 - 3.31	< 0.001			
Ki67 labeling index							
Low (≤ 20%)	62	Reference			Reference		
High (> 20%)	165	1.28	0.86 - 1.92	0.224	1.35	0.86 - 2.11	0.189
Molecular subtypes							
Luminal	181	Reference			Reference		
Luminal-HER2	32	1.44	0.89 - 2.32	0.134	1.36	0.72 - 2.58	0.349
HER2-enriched	30	1.18	0.69 - 2.01	0.545	0.74	0.37 - 1.49	0.398
Triple-negative	66	2.82	2.00 - 3.97	< 0.001	3.05	1.96 - 4.74	< 0.001
Organs of initial distant recurrence							
Bones	105	Reference			Reference		
Lungs	91	1.07	0.72 - 1.60	0.741	1.55	0.90 - 2.66	0.111
Liver	64	1.86	1.23 - 2.81	0.003	2.78	1.54 - 5.03	< 0.001
Brain	49	3.48	2.29 - 5.29	< 0.001	2.89	1.60 - 5.22	< 0.001
Primary trigger for MBC diagnosis							
Regular surveillance	172	Reference			Reference		
Subjective symptoms	123	2.17	1.59 - 2.95	< 0.001	2.13	1.30 - 3.51	0.003

HR: hazard ratio; 95% CI: 95% confidence interval; MBC: metastatic breast cancer; HER2: human epidermal growth factor receptor 2.

### Treatment patterns according to the site of initial distant recurrence

**Figure 3:**
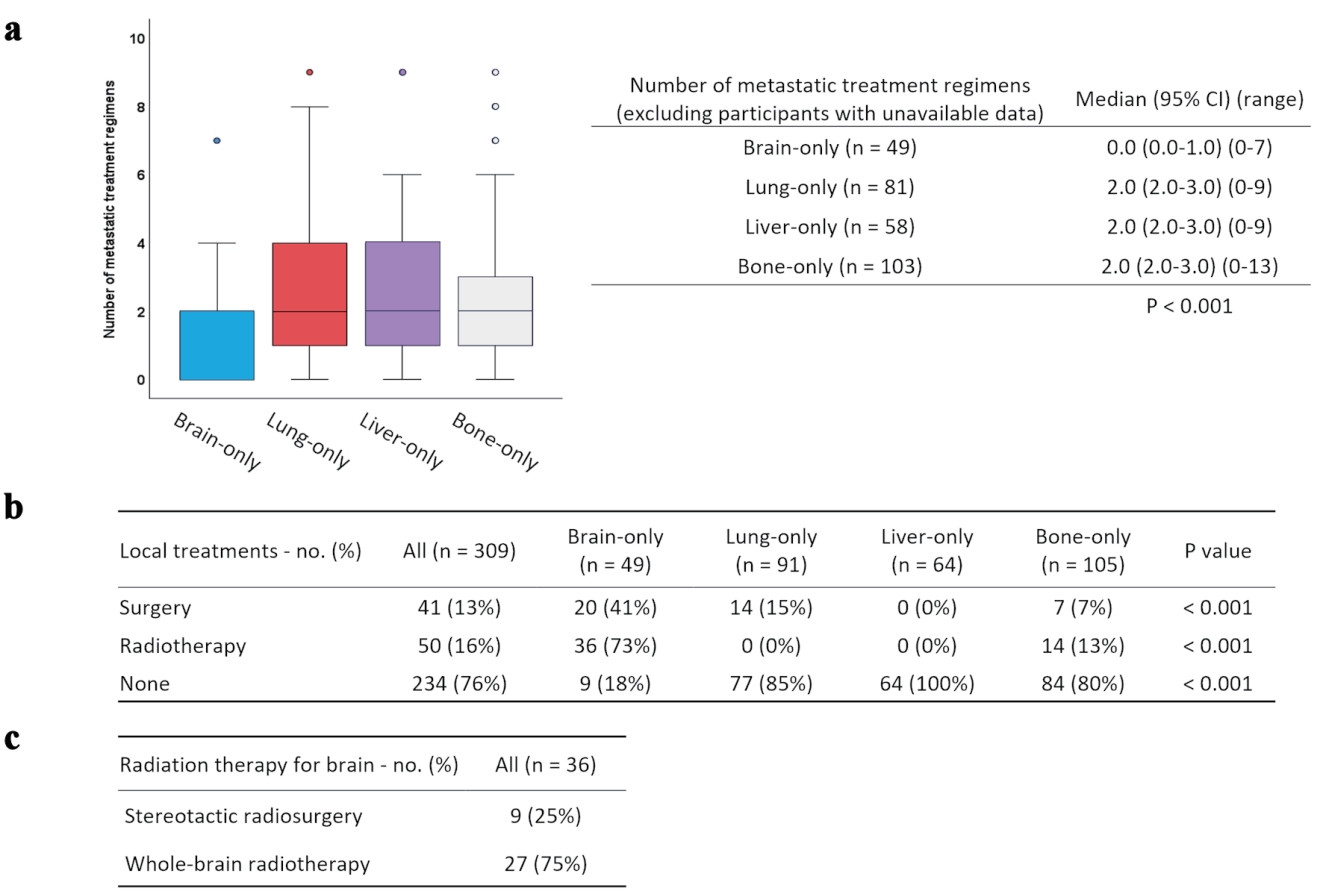
Systemic and local treatment patterns after distant recurrence according to the site of initial metastasis. (a) Number of treatment regimens for MBC according to organs of initial distant recurrence are shown and stratified by the box plots. (b) Details of local treatment for organs of initial distant recurrence. (c) Details of radiation therapy for brain metastasis.

The number of drug treatment regimens administered for MBC after the diagnosis of distant recurrence is shown in Figure 3a. Among patients for whom treatment course data were available, the number of drug regimens was significantly lower in patients with brain recurrence compared to those with recurrence in other organs (P < 0.001).

Next, details of the local treatment (surgery/radiotherapy) for the organs of initial distant recurrence are summarized in Figure 3b-c and were as follows. Local treatment was most frequently performed for brain metastases (82%), including surgery in 41% and radiotherapy in 73% of patients. Among the 36 patients who received radiotherapy for brain metastases, 25% underwent stereotactic radiosurgery, and 75% received whole-brain radiation therapy (WBRT), including cases in which WBRT was combined with surgical resection. For lung metastases, 15% of patients underwent surgery, all of which was performed for diagnostic purposes, and no radiotherapy was administered. No local treatment was performed for liver metastases. For bone metastases, surgery was performed in 7% and radiotherapy in 22% of patients, with all radiotherapy being delivered for palliative purposes.

### Site-specific prognostic factors for PRS according to the initial distant metastasis

Given the marked differences in the primary trigger for MBC diagnosis and subsequent prognosis according to the site of metastasis, we finally performed an exploratory analysis of prognostic factors stratified by the site of initial distant metastasis. As shown in Table 3, in the brain-only group, which exhibited the poorest prognosis, patients with high nuclear grade tumors tended to have shorter PRS (P = 0.064). As for molecular subtype, when luminal tumors were used as the reference, TNBC exhibited a higher HR of 1.87 (P = 0.126), although this difference did not reach statistical significance. However, when TNBC was used as the reference, the HRs for the other subtypes were as follows; luminal (HR = 0.53, P = 0.126), luminal-HER2 (HR = 0.37, P = 0.044) and HER2-enriched (HR = 0.44, P = 0.058). Among these, luminal-HER2 tumors showed a significantly longer PRS compared to TNBC. In addition, patients who did not receive local treatment for brain metastasis also tended to have shorter PRS (P = 0.057).

**Table 3:** Site-Specific Factors Relating With Post-Relapse Survival

Clinicopathological factors	Brain-only				Lung-only				Liver-only				Bone-only			
	N	HR	95% CI	P value	N	HR	95% CI	P value	N	HR	95% CI	P value	N	HR	95% CI	P value
Age at MBC diagnosis	49	1.02	0.99 - 1.05	0.139	91	1.03	1.00 - 1.05	0.033	64	1.01	0.98 - 1.03	0.652	105	1.02	0.99 - 1.04	0.154
Histological type																
Invasive ductal carcinoma	45	Reference			76	Reference			58	Reference			87	Reference		
Others	4	0.60	0.18 - 1.96	0.396	15	0.99	0.44 - 2.21	0.971	6	1.73	0.67 - 4.46	0.254	18	2.30	1.22 - 4.33	0.010
Nuclear grade																
Grade 1/2	11	Reference			33	Reference			30	Reference			67	Reference		
Grade 3	12	2.58	0.95 - 7.05	0.064	29	2.79	1.27 - 6.13	0.011	21	1.87	0.92 - 3.79	0.084	14	1.62	0.73 - 3.62	0.238
Ki67 labeling index																
Low (≤ 20%)	9	Reference			9	Reference			20	Reference			25	Reference		
High (> 20%)	25	1.11	0.47 - 2.63	0.811	64	2.35	0.72 - 7.67	0.158	29	1.87	0.85 - 4.10	0.119	46	0.85	0.41 - 1.74	0.655
Molecular subtypes																
Luminal	14	Reference			47	Reference			39	Reference			81	Reference		
Luminal - HER2	7	0.69	0.25 - 1.90	0.476	7	1.77	0.60 - 5.24	0.304	11	0.86	0.35 - 2.10	0.738	7	1.42	0.50 - 4.02	0.507
HER2 - enriched	13	0.82	0.34 - 1.98	0.654	11	0.77	0.26 - 2.32	0.642	3	0.48	0.06 - 3.52	0.467	3	1.58	0.38 - 6.62	0.531
Triple - negative	15	1.87	0.84 - 4.18	0.126	26	3.37	1.75 - 6.48	< 0.001	11	3.19	1.46 - 6.94	0.004	14	2.29	1.15 - 4.54	0.018
Primary trigger for MBC diagnosis																
Regular surveillance	2	Reference			75	Reference			46	Reference			49	Reference		
Subjective symptoms	44	1.30	0.31 - 5.48	0.721	13	5.04	2.32 - 10.98	< 0.001	14	3.34	1.53 - 7.27	0.002	52	1.37	0.77 - 2.45	0.290
Local treatment for initial MBC																
Yes	40	Reference			14	Reference			0	Reference			21	Reference		
No	9	2.16	0.98 - 4.78	0.057	77	2.95	1.05 - 8.27	0.039	64	NA	NA	NA	84	0.45	0.25 - 0.83	0.011

HR: hazard ratio; 95% CI: 95% confidence interval; MBC: metastatic breast cancer; HER2: human epidermal growth factor receptor 2; NA: not available.

In the lung-only group, shorter PRS was observed among older patients and those with high nuclear grade tumors, TNBC, or symptomatic presentation at the time of diagnosis of metastasis (P = 0.033, P = 0.011, P < 0.001, and P < 0.001, respectively). Interestingly, patients who underwent local surgery had significantly better PRS (P = 0.039); however, all surgical procedures were performed for diagnostic purposes only. Among patients who developed liver metastasis, those with TNBC or who presented with symptoms caused by liver dysfunction had significantly worse PRS (P = 0.004 and P = 0.002, respectively). Finally, in the bone-only group, patients with special histological types or TNBC had significantly worse prognosis (P = 0.010 and P = 0.018, respectively). In addition, patients who required local treatment for symptomatic bone lesions at the time of diagnosis also had significantly shorter PRS (P = 0.011).

**Figure 4:**
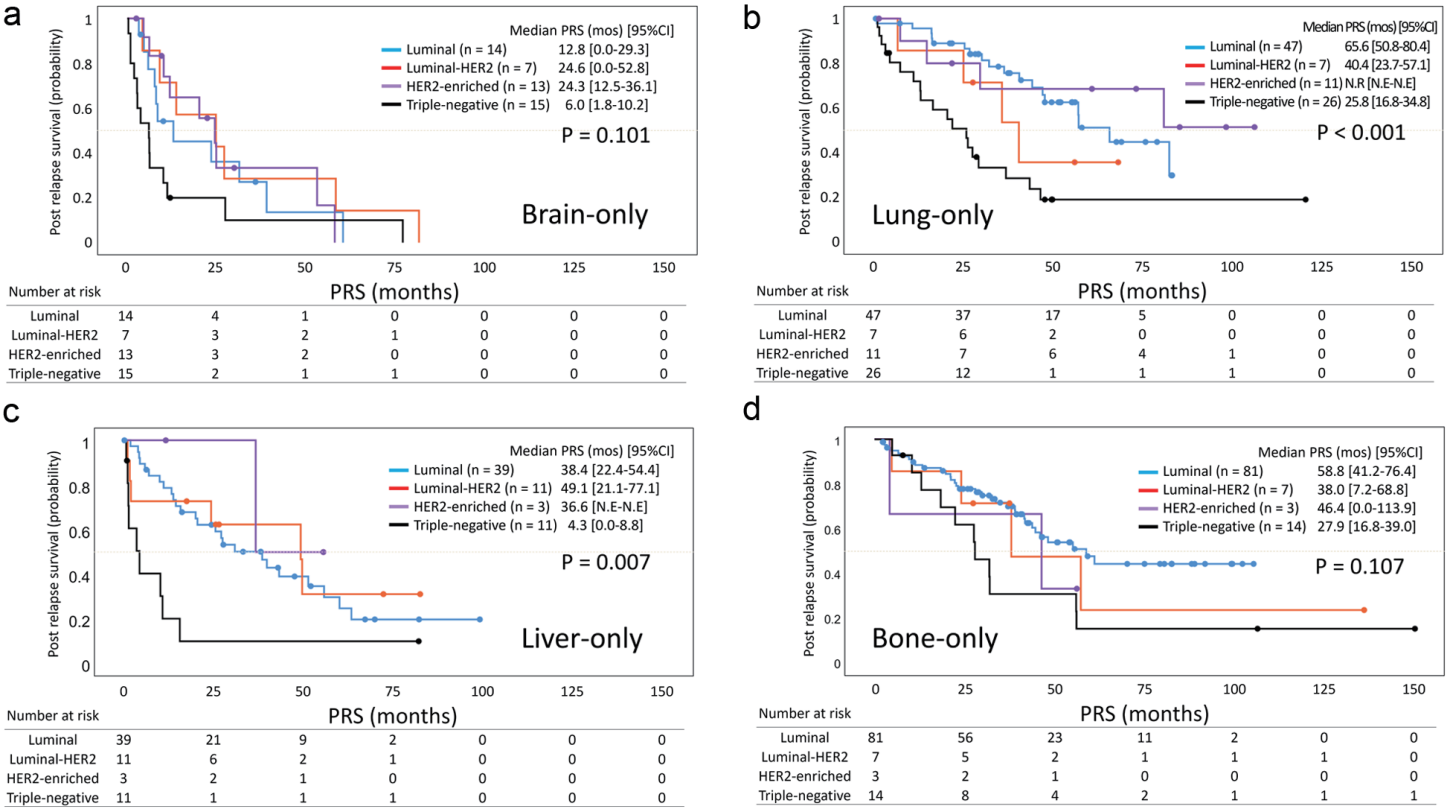
Kaplan-Meier curves of post-relapse survival by molecular subtype in each metastatic site. Kaplan-Meier curves of PRS according to molecular subtype in the (a) brain-only, (b) lung-only, (c) liver-only, and (d) bone-only groups are shown. The blue, red, purple, and black curves correspond to patients with luminal, luminal-HER2, HER2-enriched, and triple-negative MBC, respectively. Dots on each curve indicate censored cases. PRS: post-relapse survival; CI: confidence interval; N.R: not reached; N.E: not estimable; MBC: metastatic breast cancer; HER2: human epidermal growth factor receptor 2.

Lastly, we compared PRS by subtype within each metastatic organ, where molecular subtype was consistently associated with prognosis, using the Kaplan-Meier curve method. As shown in Figure 4, TNBC was consistently associated with the shortest PRS across all metastatic sites. In the lung-only and liver-only groups, significant differences were observed in the distribution of Kaplan-Meier curves, reflecting the markedly worse prognosis of TNBC compared to the more favorable outcomes in the luminal type (median PRS: lung-only group, TNBC 25.8 months vs. luminal 65.6 months; liver-only group, TNBC 4.3 months vs. luminal 38.4 months; P < 0.001 and P = 0.007, respectively).

## Discussion

In the present study, a comparison across metastatic organs revealed several distinct features. Patients with HER2-enriched tumors had a higher propensity for brain metastasis, those with TNBC more frequently developed brain and lung metastases, and bone metastasis was most commonly observed in patients with luminal-type tumors. This trend is consistent with findings from previous clinical and preclinical studies [12-14]. Moreover, DMFS varied significantly depending on the metastatic site, with a trend indicating that the timing of recurrence differed among organs: longest for bone, followed by lung, and shortest for brain.

Several previous studies have reported that patients with bone-only metastases have more favorable survival outcomes compared to those with lung-only metastasis [8, 15, 16]. Meanwhile, some reports have suggested that the prognostic difference between bone and lung metastases may be minimal, with survival curves for the two sites being relatively close [6, 7]. In the present study as well, no significant difference in PRS was observed between the bone-only and lung-only groups. This result may be partly explained by the possibility that a relatively large proportion of lung metastases were detected through routine follow-up examinations without any symptom. In addition, some lung lesions required surgical confirmation because of their small or solitary nature, which may have been associated with better prognosis in these patients. Alternatively, recent advances in systemic therapy for recurrent breast cancer may have contributed to improved prognosis in patients with lung metastases. Nevertheless, the difference in prognosis between these two groups may not be substantial. Some population-based studies have shown site-specific survival differences in single-organ metastasis, but the prognostic gap between bone-only and lung-only disease remains unclear, consistent with our finding of comparable outcomes [8, 9].

Prognostic factors associated with PRS were also explored for each metastatic organ, revealing distinct patterns in each group. TNBC was a common poor prognostic factor across all metastatic sites. In the brain metastasis group, high nuclear grade and absence of local treatment tended to be associated with worse prognosis. In the lung metastasis group, advanced age and symptom-driven diagnosis were linked to shorter PRS. In the liver group, symptomatic recurrence accompanied by hepatic dysfunction was a poor prognostic indicator, while in the bone group, tumors with special histological types and symptomatic lesions requiring local treatment were associated with unfavorable outcomes. A notable finding was that the presence of symptoms at the time of diagnosis varied across metastatic sites and was associated with prognosis. While nearly all patients with brain metastases were diagnosed based on neurological symptoms, the presence of symptoms was statistically associated with shorter PRS in both the lung-only and liver-only groups. In the bone-only group as well, poor outcomes were observed in patients who presented with advanced symptoms severe enough to require local intervention, such as pain or pathological fractures. These findings may reflect that symptomatic recurrence is often associated with more advanced metastatic disease and impaired performance status, both of which contribute to poor prognosis. As for the brain-only group, 96% of patients were diagnosed after the onset of neurological symptoms, indicating that asymptomatic detection was extremely rare and that brain metastases were often identified at an advanced stage, potentially limiting subsequent treatment options. This may also explain why local treatment could not be applied in some patients with brain metastases. Meanwhile, recent agents such as trastuzumab deruxtecan (T-DXd) have demonstrated intracranial efficacy [17-19], and their benefits may be greater if treatment is initiated earlier. Therefore, developing strategies to detect brain metastases during the asymptomatic phase, through improved risk stratification or predictive markers, may be important. Based on our findings, clinical features such as advanced initial stage, TNBC or HER2-enriched subtype, and favorable response to neoadjuvant therapy (e.g., pathological complete response), which was observed in a relatively high proportion of patients with brain metastasis in our cohort, may help identify a subset of patients who are at risk of early distant recurrence such as brain metastasis, despite favorable initial response. Additionally, noninvasive methods such as liquid biopsy may offer promising tools for early detection in the future.

Major limitations in the current study include its retrospective design based on real-world data, which raises concerns about potential selection bias across metastatic site groups. This bias may arise from differences in the detectability of metastases depending on postoperative surveillance strategies. For instance, metastases to organs such as the lungs or liver are more likely to be detected through routine imaging or laboratory monitoring, whereas brain metastases are often identified only after the onset of symptoms. In addition, the methods of postoperative surveillance and treatment strategies for MBC may have varied, as follow-up schedules were determined by each physician’s discretion, reflecting real-world clinical practice in Japan. Second, this study focused on patients with initial distant recurrence limited to a single organ and excluded those with multi-organ metastases. However, information to determine whether these lesions represented true oligometastatic disease was unavailable, which prevented a more detailed evaluation of its prognostic implications. Another limitation is that aggressive special forms of metastasis regarded as rapidly progressive variants within each organ, such as liver crisis in liver metastasis, were excluded from this analysis. Although this exclusion aimed to ensure comparability of disease burden across metastatic sites, it may have resulted in the underrepresentation of highly aggressive disease phenotypes. Furthermore, for the included patients, detailed information on post-recurrence disease progression or additional organ metastases could not be obtained. Such information would be important for evaluating whether the prognosis is determined mainly by the initial metastatic site or by subsequent disease evolution, as demonstrated in previous large-scale studies [8, 20]. Future prospective studies with longitudinal follow-up are warranted to address this issue.

Nevertheless, to the best of our knowledge, no other large-scale studies have focused specifically on the biology of MBC in Asian populations, examining initial distant recurrence to single, anatomically distinct organs. A key strength of this study lies in its strict inclusion of patients with single-organ metastasis, which enabled a focused comparison of the biological characteristics and clinical features associated with metastasis to each organ. Previous studies investigating single-organ metastasis have mostly been conducted in Western populations, whereas our results, derived from a Japanese cohort, generally aligned with these findings. This suggests that the prognostic impact of metastatic organ site may be broadly consistent across different ethnic and healthcare settings.

### Conclusions

Based on a single-organ metastasis cohort, this study demonstrated that MBC exhibits distinct prognostic patterns depending on the site of initial distant recurrence. Particularly, brain metastasis was associated with markedly poor outcomes, likely due to advanced disease status at diagnosis and limited therapeutic options. These findings may serve as a foundation for future efforts in risk stratification and treatment planning for recurrent breast cancer.

## Supplementary Material

Suppl 1 Kaplan-Meier curves for distant metastasis-free survival according to the site of initial distant metastasis.

## Data Availability

All data supporting the findings of this study are available within the paper and its supplementary material.

## Abbreviations

ABC: advanced breast cancer
CI: confidence interval
DMFS: distant metastasis-free survival
ER: estrogen receptor
HER2: human epidermal growth factor receptor 2
MBC: metastatic breast cancer
PR: progesterone receptor
PRS: post-relapse survival
SEER: Surveillance, Epidemiology, and End Results
T-DXd: trastuzumab deruxtecan
TNBC: triple-negative breast cancer
WBRT: whole-brain radiation therapy
