# Supplementary material for: Organ-Specific Clinicopathological Features That Are Associated With Post-Relapse Survival of Metastatic Breast Cancer in Japanese Women: A Multicenter Cohort Study
Source: World J Oncol. 2025 Dec 17;17(1):52–62. doi: 10.14740/wjon2662 (PMC12758093; doi:10.14740/wjon2662)
Supplement: Suppl 1 — Kaplan-Meier curves for distant metastasis-free survival according to the site of initial distant metastasis. [file wjon-17-01-052-s001.docx]

**Suppl 1.** Kaplan–Meier curves for distant metastasis-free survival according to the site of initial distant metastasis.

**
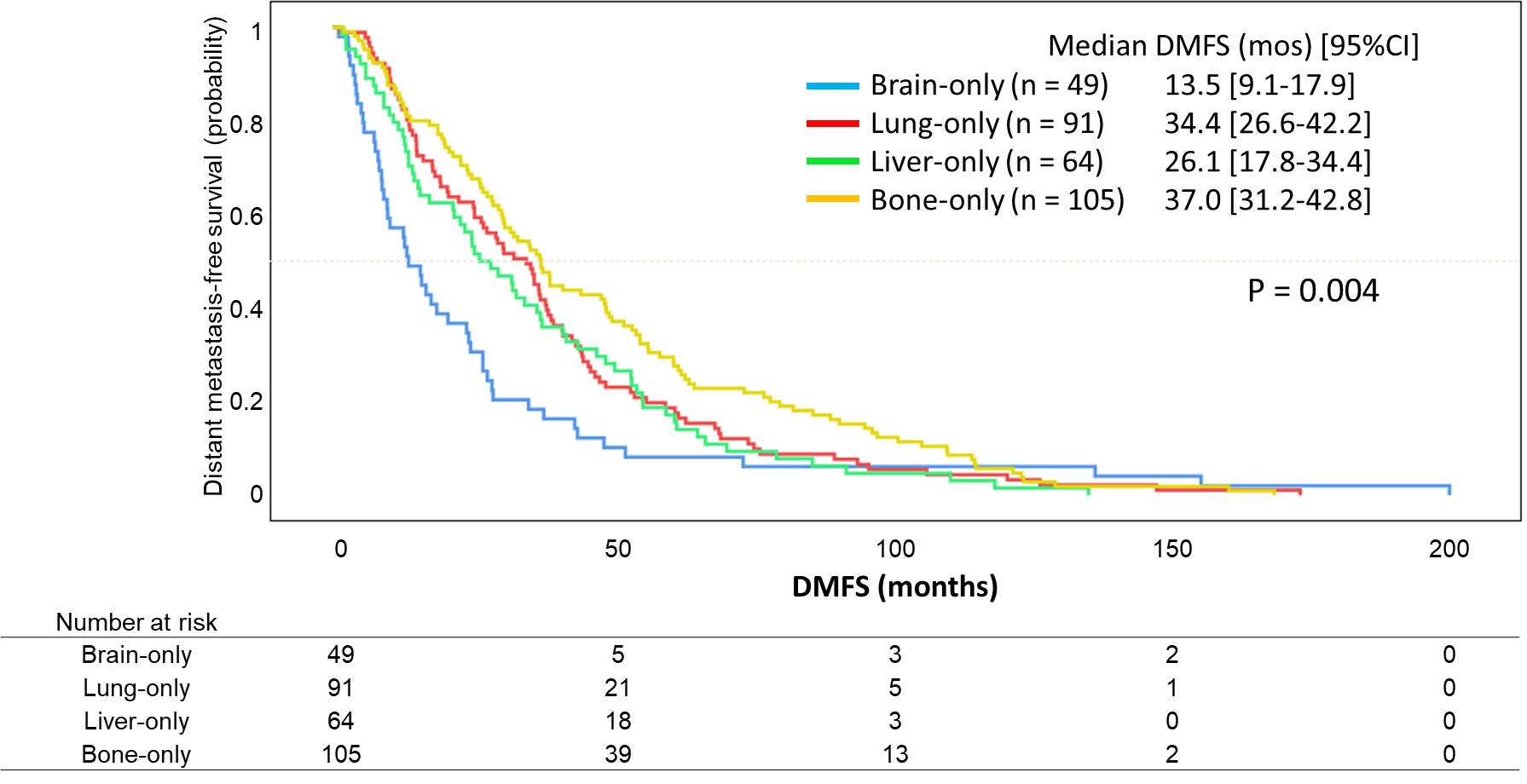
**
